# An integrated SMC–NADRC robust control approach for electric power steering systems considering nonlinear friction and parametric uncertainties

**DOI:** 10.1371/journal.pone.0346332

**Published:** 2026-04-09

**Authors:** Tuan Anh Nguyen, Duc Ngoc Nguyen

**Affiliations:** Automotive Engineering Division, Thuyloi University, Kim Lien, Hanoi, Vietnam; Buckinghamshire New University - High Wycombe Campus: Buckinghamshire New University, UNITED KINGDOM OF GREAT BRITAIN AND NORTHERN IRELAND

## Abstract

Electric Power Steering (EPS) facilitates easier steering and has become widely equipped in modern vehicles. Many prior studies on EPS control have tended to overlook the effects of nonlinear friction and parametric uncertainties, while traditional control strategies often suffer from overshooting, phase lag, and chattering. This article proposes a nonlinear robust control scheme that integrates smooth Sliding Mode Control (SMC) with Nonlinear Active Disturbance Rejection Control (NADRC) to address these challenges. The influence of nonlinearities and uncertainties is compensated in the control law through an augmented variable estimated by a Nonlinear Extended State Observer (NESO), and the reference signal is smoothed by a Nonlinear Tracking Differentiator (NTD). Simulation results demonstrate that the Root Mean Square (RMS) tracking errors of the steering column angle and steering motor angle are only 0.165% and 0.172%, respectively, when the proposed approach controls the system without considering nonlinear friction and parameter uncertainties. When both adverse factors are included, the tracking errors increase only slightly to 0.704% and 0.736%. Moreover, the proposed method achieves a total energy consumption of just 1194.581 J, which is lower than that of the compared controllers. In addition, the effects of overshooting, phase delay, and chattering are eliminated. Overall, the proposed control shows strong potential for improving the performance of automotive mechatronic systems.

## 1. Introduction

The steering system maintains and controls the vehicle’s direction of motion. In practice, steering can be demanding because the driver must generate sufficient hand torque on the steering wheel to overcome both the road resistance and the system’s internal frictional torque. Most modern vehicles have Electric Power Steering (EPS), significantly reducing steering effort and improving the steering feel [1]. Compared to conventional power-assisted steering mechanisms, EPS provides several distinct advantages, including compactness, ease of integration, high responsiveness, and environmental friendliness [2]. According to Ramasamy [3], fuel efficiency can be improved by approximately 3% when replacing a hydraulic steering system with a modern EPS unit. Furthermore, EPS can be readily integrated into electric, hybrid, and autonomous vehicles [4].

A wide range of control strategies has been applied to improve the performance of EPS systems. In [5], Hassan et al. developed a Proportional–Integral–Derivative (PID) controller whose gains were optimally tuned using a binary Genetic Algorithm (GA) to minimize motor current error. Zheng and Wei [6] introduced the application of fuzzy logic for phase compensation through PID parameter adjustment. Simulation results demonstrated that current tracking was improved by 75.2%, while overshooting was reduced by 72.9%. Hanifah et al. [7] compared a conventional PID and a PID tuned by Ant Colony Optimization (ACO), with experimental findings showing a slight decrease in peak motor current from 25.02 A to 24.99 A. The use of a PID–PI control strategy to maintain EPS stability was presented by Manca et al. [8]; however, phase delay was seen in the identified model response during sine-sweep maneuvers. Regarding heavy-duty vehicles requiring high assistance torque, integrating two motors independently controlled by separate PID controllers has been considered necessary [9,10]. Additional fuzzy logic and neural network applications for PID tuning have also been reported in [11,12]. Despite these efforts, PID controllers exhibit notable drawbacks when dealing with complex systems. Simulation results in [13] revealed that the overshooting in the step response of PID was substantially higher than that achieved with H_∞_ control. This conclusion was further supported by the analytical results reported by Zhao and Zhang [14]. Moreover, the PID approach primarily applies to single-input and single-output systems.

Chitu et al. [15] designed a Linear Quadratic Regulator (LQR) controller for the performance control of EPS systems. Their approach was built upon a discrete-time model in which the weighting matrices were required to be positive definite or semi-definite. Irmer and Henrichfreise [16] designed a robust Linear Quadratic Gaussian (LQG) compensator based on the combination of LQR and LQ Estimation (LQE) to control the EPS system. However, the torsion bar torque error is significant. A Fault-Tolerant Control (FTC) based on LQR optimization theory was presented in [17] by Liu et al. Nguyen [18] designed LQ Tracking (LQT) control to improve the tracking error of EPS systems based on the correction of inputs through fuzzy logic systems. Nevertheless, control mechanisms derived from LQR rely on the full availability of system states. When physical sensors are used for measurement, noise may arise, leading to performance degradation [19].

Several applications of Backstepping Control (BSC) for improving EPS performance have been introduced recently. Shi et al. [20] presented a Dynamic Range Control (DRC) strategy based on a BSC framework for electrohydraulic steering systems. A hybrid approach combining BSC and PID, where two component control signals were synthesized, was presented in [21]. However, the issue of global system stability was not fully addressed. In [22], Nguyen proposed an integrated control structure in which the input of the BSC was adjusted by a PID controller whose parameters were adaptively tuned using a fuzzy logic system. Additional applications of fuzzy-based BSC for EPS control were discussed in [23–25]. Despite these advances, the design of BSC remains challenging, particularly in selecting appropriate virtual control variables and simplifying the system model, both of which may contribute to increased errors in practical implementations.

Disturbances have a significant influence on EPS control. In [26], Murilo et al. assumed that rectangular pulses could represent road resistance torque to simplify the computational process. Similarly, Iqbal and Nguyen [27] modeled external disturbances as sinusoidal excitations. However, these may lead to considerable inaccuracies when applied to real-world scenarios. To address this limitation, Na et al. [28] developed an Active Disturbance Rejection Control (ADRC) scheme to mitigate the effects of external disturbances and improve steering wheel torque tracking. The state feedback law was formulated based on a PI controller in their design. While the angular signal followed its reference with only minor error, the angular velocity, which is smoothed by the tracking differentiator, exhibited significant oscillations. Ma et al. [29] extended ADRC to a physical test bench. However, the absence of a comparison with other controllers made it challenging to validate the practical performance. Zheng and Wei [30] later evaluated ADRC against conventional PID and Fuzzy PID under harsh EPS operating conditions. Their simulation results showed that the adjustment time was reduced to 0.237 s, while overshoot decreased to 1.3%. Although ADRC provides disturbance rejection and uncertainty compensation through an Extended State Observer (ESO), its control law often remains dependent on a conventional PID framework, which may limit performance under heavy-duty conditions.

Sliding Mode Control (SMC) has been widely utilized for its effectiveness in handling complex systems. In [31], Kim et al. introduced a traditional SMC combined with a Disturbance Observer (DOB) to control a Column-type EPS (CEPS) system. Khasawneh and Das [32] designed a robust SMC framework that explicitly accounted for the nonlinear friction effects in the steering motor angle. However, calculation results showed that chattering in the pinion angle remained considerable. Lee et al. [33] proposed an adaptive SMC to improve steering wheel torque tracking. However, the simulations revealed significant chattering in steering wheel speed and acceleration. In [34], Lu et al. incorporated a fuzzy logic system into the adaptive SMC structure to address this issue. At the same time, a related approach utilized an adaptive fuzzy surface within the SMC scheme to further reduce chattering and improve EPS tracking accuracy [35]. Nguyen [36] combined SMC, PID, and BSC to construct a robust controller controlling the EPS system. Although the outputs closely followed the references, the algorithm structure was highly complex, which raises concerns for real-time implementation. Overall, SMC-based designs consistently encounter the challenge of chattering. Moreover, parameter uncertainties and nonlinearities can further degrade system performance if such effects are not explicitly compensated within the control law.

Various observers have been proposed to estimate nonlinear effects and parameter uncertainties in EPS systems. Early work by Lee et al. [37] introduced a linear disturbance observer expressed in transfer function form, while an adaptive version was later designed to account for crosswind effects [38]. Kalman filtering has also been employed for disturbance estimation [39], although its use is largely restricted to cases where Gaussian noise assumptions hold, as noted by Li et al. [40]. Beyond these methods, Yamamoto [41] proposed an H_∞_/PI observer for estimating driver torque, and Kim et al. [42] applied a linear Extended State Observer (LESO) to capture parameter variations in a nonlinear EPS model. Other observer-based designs for EPS can be found in [43,44]. Despite these contributions, most existing methods remain limited by assumptions on disturbance characteristics or by the simplified nature of the system models.

### Research gaps and key contributions

The above literature review reveals several unresolved issues in existing EPS control strategies. First, conventional SMC provides strong robustness but often suffers from chattering and performance degradation when unmodeled dynamics and parameter variations are present. Second, ADRC–based approaches rely heavily on observer accuracy and typically employ PID-type control laws, which may introduce phase lag and limit performance under heavy-duty steering conditions. Third, most existing studies do not explicitly distinguish between robustness enforcement and uncertainty compensation, leading to complex control structures or limited adaptability in practical EPS systems.

To address these gaps, this article proposes an integrated robust control framework that combines smooth SMC with Nonlinear Active Disturbance Rejection Control (NADRC). In the proposed structure, SMC is employed as the primary tracking mechanism to guarantee robustness and fast convergence. At the same time, a Nonlinear Extended State Observer (NESO) is used to estimate and compensate for lumped uncertainties and nonlinear effects in real time. This separation between robustness enforcement and uncertainty compensation constitutes the main contribution of the present work. In addition, a Nonlinear Tracking Differentiator (NTD) is incorporated to smooth reference signals, and a smooth nonlinear function replaces the discontinuous switching term in conventional SMC to eliminate chattering. The effectiveness of the proposed approach is demonstrated through comparative simulations under both nominal and uncertain operating conditions.

The article is organized into four sections. The Introduction outlines the literature review, research gaps, and the key contributions. The second section describes system modeling and control design. Simulation results are analyzed and discussed in the third section. Finally, key findings and potential directions for future development are presented in the Conclusion section.

## 2. Mathematical models

This section presents the system’s dynamic modeling and the control mechanism’s design.

### 2.1. Dynamic model

**Fig 1:**
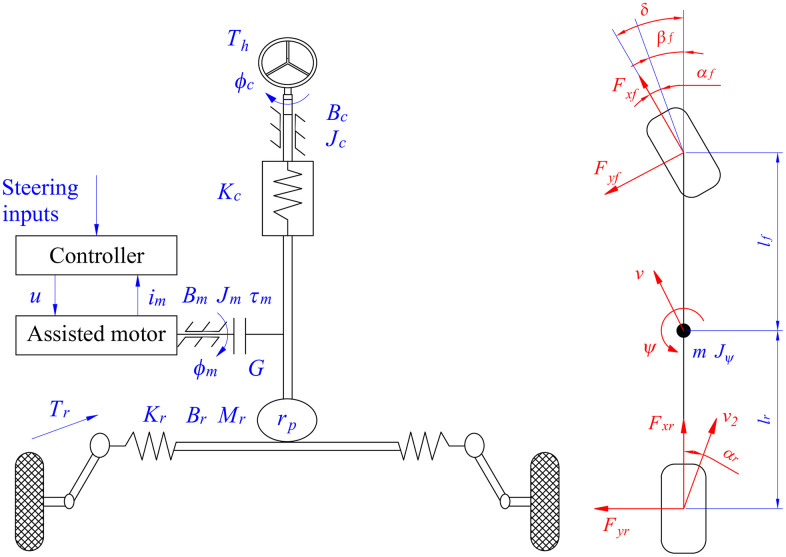
EPS system model. (a) EPS system model (b) Vehicle dynamics model.

Fig 1 illustrates the structure of a typical CEPS system, which consists of a steering column, a steering wheel, a motor gear, a rack and pinion, an assisted motor, and sensors.

Equations (1) to (3) illustrate the ideal dynamics of the EPS system [45], where *J*_*c*_ is steering column inertia, *B*_*c*_ is steering column damping, *K*_*c*_ is steering column stiffness, *T*_*h*_ is hand driver, *G* is motor gear ratio, *φ*_*c*_ is steering column angle, *r*_*p*_ is pinion radius, *φ*_*m*_ is steering motor angle, *K*_*t*_ is torque parameter, *i*_*m*_ is motor current, *u* is control input, *L*_*m*_ is motor inductance, *R*_*m*_ is motor resistance, and *T*_*r*_ is road resistance torque.


$$J_{c}{\ddot{\phi }}_{c}+B_{c}{\dot{\phi }}_{c}+K_{c}\left(\phi_{c}-\frac{\phi_{m}}{G}\right)=T_{h}$$ (1)

$$J_{eq}{\ddot{\phi }}_{m}+B_{eq}{\dot{\phi }}_{m}+\frac{K_{c}+K_{r}\overset{\text{2}}{\underset{p}{r}}}{G^{\text{2}}}\phi_{m}=\frac{K_{c}}{G}\phi_{c}+K_{t}i_{m}-\frac{T_{r}}{G}$$ (2)

$$K_{t}{\dot{\phi }}_{m}+L_{m}{\dot{i}}_{m}+R_{m}i_{m}=u$$ (3)

Equivalent damping (*B*_*eq*_) and equivalent inertia (*J*_*eq*_) are determined according to (4) and (5), respectively, where *B*_*m*_ is the steering motor damping, *B*_*r*_ is the rack damping, *J*_*m*_ is the steering motor inertia, and *M*_*r*_ is the rack mass.


$$B_{eq}=B_{m}+B_{r}\frac{\overset{\text{2}}{\underset{p}{r}}}{G^{\text{2}}}$$ (4)

$$J_{eq}=J_{m}+M_{r}\frac{\overset{\text{2}}{\underset{p}{r}}}{G^{\text{2}}}$$ (5)

The ideal model presented above is used to generate the reference signal. In practice, nonlinear friction and parametric uncertainties often affect the system dynamics. As a result, equation (2) is rewritten as (6), where *B*_*eq0*_ is the nominal value of equivalent damping, Δ*B*_*eq*_ is the parametric uncertainty, and *τ*_*m*_ is the coulomb friction coefficient.


$$J_{eq}{\ddot{\phi }}_{m}+\left(B_{eq0}+\Delta B_{eq}\right){\dot{\phi }}_{m}+\tau_{m}tanh\left({\dot{\phi }}_{m}\right)+\frac{K_{c}+K_{r}\overset{\text{2}}{\underset{p}{r}}}{G^{\text{2}}}\phi_{m}=\frac{K_{c}}{G}\phi_{c}+K_{t}i_{m}-\frac{T_{r}}{G}$$ (6)

Although the EPS system is pre-designed, several parameters cannot be regarded as perfectly known or constant in practice. Mechanical parameters such as equivalent damping and inertia may vary over time due to temperature changes, component wear, and lubrication conditions. Electrical parameters of the assist motor, including resistance and torque constant, are also affected by thermal effects and aging. In addition, nonlinear phenomena such as Coulomb friction and unmodeled dynamics cannot be accurately captured by the nominal model. These effects are therefore treated as parametric uncertainties and internal nonlinearities in the proposed framework.

Road resistance torque is generated during steering, representing the interaction of the tire with the road surface. According to [46], *T*_*r*_ depends on the lateral tire force (*F*_*y*_) and is defined in (7), where *γ*_*k*_ is the kingpin angle, *γ*_*c*_ is the caster angle, *l*_*n*_ is the arm length, and *l*_*c*_ is the caster trail.


$$T_{r}\approx r_{p}l_{c}\frac{cos^{\text{2}}\left(\gamma_{c}\right)cos^{\text{2}}\left(\gamma_{k}\right)}{l_{n}}F_{y1}$$ (7)

The variation in lateral tire force is determined through a linear vehicle dynamics model (Fig 1) according to equations (8), (9), and(10), where *m* is vehicle mass, *v*_*x*_ is longitudinal speed, *v*_*y*_ is lateral speed, *F*_*x*_ is longitudinal tire force, *δ* is steering angle, *ψ* is yaw angle, *J*_*ψ*_ is yaw inertia, *l*_*f*_ is distance from center to front axle, and *l*_*r*_ is distance from center to rear axle.


$$m\left({\dot{v}}_{x}-\dot{\psi }v_{y}\right)=F_{xf}\text{ cos }\delta +F_{xr}-F_{yf}\;sin\;\delta$$ (8)

$$m\left({\dot{v}}_{y}+\dot{\psi }v_{x}\right)=F_{yf}\text{ cos }\delta +F_{yr}+F_{xf}\;sin\;\delta$$ (9)

$$J_{\psi }\ddot{\psi }=l_{f}\left(F_{xf}\;sin\;\delta +F_{yr}\text{ cos }\delta \right)-l_{r}F_{yr}$$ (10)

As mentioned above, the ideal model is used to generate the reference signals. The ideal assisted torque (*T*_*a_ideal*_) is defined according to (11), where *k*_*g*_ and *k*_*a*_ are curve parameters, ***e*** is Euler’s number, and *k*_*hi*_ are coefficients.


$$T_{a}\left(v,T_{h}\right)=k_{g}\left(k_{h1}v^{\text{2}}+k_{h2}v+k_{h3}\right)\left(\frac{\text{1}}{1+e^{-k_{a}\overset{\text{3}}{\underset{h}{T}}}}-\frac{\text{1}}{1+e^{k_{a}\overset{\text{3}}{\underset{h}{T}}}}\right)$$ (11)

**Fig 2:**
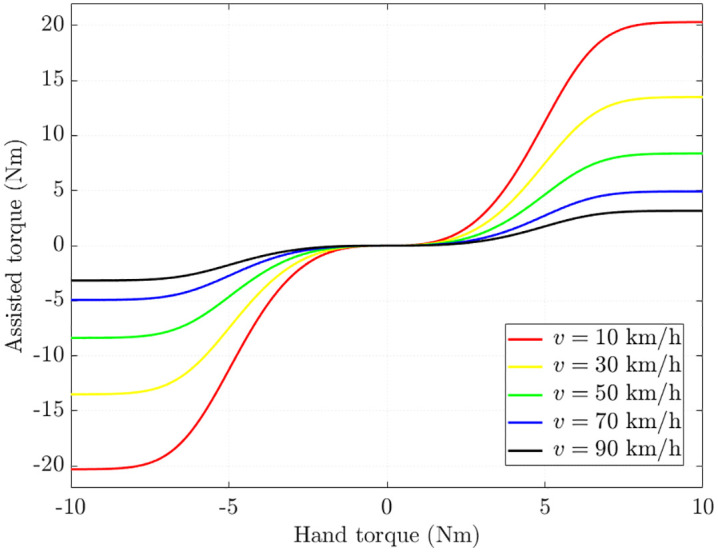
Ideal assisted torque map.

Equation (11) indicates that the ideal assisted torque depends on both vehicle speed (*v*) and hand torque (*T*_*h*_). Their relationship is illustrated in Fig 2 [19]. A closer examination of this figure reveals that the assisted torque is nearly zero when the hand torque is minimal. As the hand torque increases, the assisted torque also rises nonlinearly, eventually approaching a saturation asymptote limit when the hand torque becomes excessively large. At the same time, the assisted torque decreases with increasing vehicle speed, which is consistent with the fundamental requirements of vehicle dynamics.

### 2.2. Control design

In this article, we propose designing a high-performance integrated control mechanism, which combines smooth SMC and NADRC to control the system performance and eliminate the influence of chattering, nonlinearity, and parameter uncertainties. Proposed control is significantly improved compared to the previous version [19].

The state variables are listed in order in (12).


$$\begin{array}{c} \left[\begin{array}{ccccc} @ccccc@x_{\text{1}} & x_{\text{2}} & x_{\text{3}} & x_{\text{4}} & x_{\text{5}} \end{array}\right]=\left[\begin{array}{ccccc} @ccccc@\phi_{c} & {\dot{\phi }}_{c} & \phi_{m} & {\dot{\phi }}_{m} & i_{m} \end{array}\right] \end{array}$$ (12)

Taking the derivatives of the state variables according to the actual model, we get (13) to (17).


$${\dot{x}}_{\text{1}}=x_{\text{2}}$$ (13)

$${\dot{x}}_{\text{2}}=-\frac{K_{c}}{J_{c}}x_{\text{1}}-\frac{B_{c}}{J_{c}}x_{\text{2}}+\frac{K_{c}}{J_{c}G}x_{\text{3}}+\frac{T_{h}}{J_{c}}$$ (14)

$${\dot{x}}_{\text{3}}=x_{\text{4}}$$ (15)

$${\dot{x}}_{\text{4}}=\frac{K_{c}}{J_{eq}G}x_{\text{1}}-\frac{K_{c}+K_{r}\overset{\text{2}}{\underset{p}{r}}}{J_{eq}G^{\text{2}}}x_{\text{3}}-\left(\frac{B_{eq0}+\Delta B_{eq}}{J_{eq}}\right)x_{\text{4}}-\tau_{m}\;tanh\left(x_{\text{4}}\right)+\frac{K_{t}}{J_{eq}}x_{\text{5}}-\frac{T_{r}}{J_{eq}G}$$ (16)

$${\dot{x}}_{\text{5}}=-\frac{K_{t}}{L_{m}}x_{\text{4}}-\frac{R_{m}}{L_{m}}x_{\text{5}}+\frac{\text{1}}{L_{m}}u$$ (17)

A Nonlinear Extended State Observer (NESO) is designed to estimate the variations arising from nonlinearities and parameter uncertainties, while mitigating the influence of sensor noise, which originates from the direct measurement of signals by physical sensors. The observed error (*e*_*o*_) is defined in (18).


$$e_{o}=x_{\text{1}}-{\hat{x}}_{\text{1}}$$ (18)

The observed state variables are rewritten according to equations (19) to (24).


$${\dot{\hat{x}}}_{\text{1}}={\hat{x}}_{\text{2}}+\lambda_{\text{1}}$$ (19)

$${\dot{\hat{x}}}_{\text{2}}=-\frac{K_{c}}{J_{c}}{\hat{x}}_{\text{1}}-\frac{B_{c}}{J_{c}}{\hat{x}}_{\text{2}}+\frac{K_{c}}{J_{c}G}{\hat{x}}_{\text{3}}+\frac{T_{h}}{J_{c}}+\lambda_{\text{2}}$$ (20)

$${\dot{\hat{x}}}_{\text{3}}={\hat{x}}_{\text{4}}+\lambda_{\text{3}}$$ (21)

$${\dot{\hat{x}}}_{\text{4}}=\frac{K_{c}}{J_{eq}G}{\hat{x}}_{\text{1}}-\frac{K_{c}+K_{r}\overset{\text{2}}{\underset{p}{r}}}{J_{eq}G^{\text{2}}}{\hat{x}}_{\text{3}}-\frac{B_{eq0}}{J_{eq}}{\hat{x}}_{\text{4}}+\frac{K_{t}}{J_{eq}}{\hat{x}}_{\text{5}}-\frac{\text{1}}{J_{eq}}{\hat{x}}_{\text{6}}+\lambda_{\text{4}}$$ (22)

$${\dot{\hat{x}}}_{\text{5}}=-\frac{K_{t}}{L_{m}}{\hat{x}}_{\text{4}}-\frac{R_{m}}{L_{m}}{\hat{x}}_{\text{5}}+\frac{\text{1}}{L_{m}}u+\lambda_{\text{5}}$$ (23)

$${\dot{\hat{x}}}_{\text{6}}=\lambda_{\text{6}}$$ (24)

In this work, uncertainties are categorized into three groups. External disturbances mainly arise from road resistance torque caused by tire–road interaction. Parametric uncertainties include variations in mechanical and electrical parameters such as equivalent damping, inertia, and motor constants. Internal nonlinearities consist of Coulomb friction and unmodeled dynamics. All these effects are lumped into a single augmented variable and estimated online using the proposed NESO, allowing unified compensation within the control law. According to (25), *x*_6_ is the augmented variable employed to estimate the effects of nonlinearity, parameter uncertainties, and road resistance torque.


$${\hat{x}}_{\text{6}}=\frac{T_{r}}{G}+\tau_{m}tanh\left(x_{\text{4}}\right)+\Delta B_{eq}x_{\text{4}}$$ (25)

The observed gains (*λ*_*i*_) are defined in (26) as the product of the nonlinear function and the observed parameters (*L*_*i*_).


$$\lambda_{i}=L_{i}\text{fal}\left(e_{o};\beta_{i};\varphi_{i}\right)$$ (26)

The nonlinear function that constitutes the observed gains is described in (27), where *β*_*i*_ are tuning coefficients and *ϕ*_*i*_ are threshold coefficients. The proposed nonlinear function can provide more accurate observed results than conventional linear functions.


$$\begin{array}{c} \text{fal}\left(e_{o};\beta_{i};\varphi_{i}\right)=\left\{ \begin{array}{cc} @cc@\frac{e_{o}}{\overset{1-\beta_{i}}{\underset{i}{\varphi }}} & \left|e_{o}\right|\leq \varphi_{i}\\ \text{sgn}\left(e_{o}\right){\left|e_{o}\right|}^{\beta_{i}} & \left|e_{o}\right|>\varphi_{i} \end{array}\right. \end{array}$$ (27)

A Nonlinear Tracking Differentiator (NTD) is designed to smooth the reference signal. Equations (28) and (29) provide details of the proposed NTD, where *k*_*v*1_, *k*_*v*2_, and *θ* are positive coefficients.


$${\dot{v}}_{\text{1}}=v_{\text{2}}$$ (28)

$${\dot{v}}_{\text{2}}=-k_{v1}r_{\text{1}}-k_{v2}\frac{r_{\text{2}}}{\sqrt{\overset{\text{2}}{\underset{\text{2}}{r}}+\theta^{\text{2}}}}+{\ddot{x}}_{3\_ref}=v_{\text{3}}$$ (29)

*r*_1_ and *r*_2_ are the errors of NTD and are explained in (30) and (31).


$$r_{\text{1}}=v_{\text{1}}-x_{3\_ref}$$ (30)

$$r_{\text{2}}={\dot{r}}_{\text{1}}=v_{\text{2}}-{\dot{x}}_{3\_ref}$$ (31)

Taking the derivative of *r*_2_ and combining it with (29), we get (32).


$${\dot{r}}_{\text{2}}=-k_{v1}r_{\text{1}}-k_{v2}\frac{r_{\text{2}}}{\sqrt{\overset{\text{2}}{\underset{\text{2}}{r}}+\theta^{\text{2}}}}$$ (32)

Control error (*e*) is defined according to (33).


$$e=v_{\text{1}}-{\hat{x}}_{\text{3}}$$ (33)

Taking the derivative of the control error, we get (34) and (35).


$$\dot{e}={\dot{v}}_{\text{1}}-{\dot{\hat{x}}}_{\text{3}}=v_{\text{2}}-{\hat{x}}_{\text{4}}-\lambda_{\text{3}}$$ (34)

$$\ddot{e}={\dot{v}}_{\text{2}}-{\dot{\hat{x}}}_{\text{4}}-{\dot{\lambda }}_{\text{3}}=z_{\text{3}}-\sum_{i=1}^{\text{6}}a_{i}{\hat{x}}_{i}-\lambda_{\text{4}}-{\dot{\lambda }}_{\text{3}}$$ (35)

where,


$$a_{\text{1}}=\frac{K_{c}}{J_{eq}G}$$ (36)

$$a_{\text{2}}=0$$ (37)

$$a_{\text{3}}=-\frac{K_{c}+K_{r}\overset{\text{2}}{\underset{p}{r}}}{J_{eq}G^{\text{2}}}$$ (38)

$$a_{\text{4}}=-\frac{B_{eq0}}{J_{eq}}$$ (39)

$$a_{\text{5}}=\frac{K_{t}}{J_{eq}}$$ (40)

$$a_{\text{6}}=-\frac{\text{1}}{J_{eq}}$$ (41)

The objective of the control problem is to regulate the steering motor angle, denoted as y in (42).


$$y={\hat{x}}_{\text{3}}$$ (42)

Taking the derivative of *y*, we get (43), (44), and (45).


$$\dot{y}={\hat{x}}_{\text{4}}+\lambda_{\text{3}}$$ (43)

$$\ddot{y}=\sum_{i=1}^{\text{6}}a_{i}{\hat{x}}_{i}+\lambda_{\text{4}}+{\dot{\lambda }}_{\text{3}}$$ (44)

$$\overset{...}{y}=\sum_{i=1}^{\text{6}}b_{i}{\hat{x}}_{i}+\sum_{i=1}^{\text{6}}a_{i}\lambda_{i}+{\dot{\lambda }}_{\text{4}}+{\ddot{\lambda }}_{\text{3}}+\underset{b}{\underbrace{\frac{K_{t}}{J_{eq}L_{m}}}}u=\hat{d}+bu$$ (45)

where,


$$b_{\text{1}}=-\frac{B_{eq0}K_{c}}{\overset{\text{2}}{\underset{eq}{J}}G}$$ (46)

$$b_{\text{2}}=\frac{K_{c}}{J_{eq}G}$$ (47)

$$b_{\text{3}}=\frac{B_{eq0}\left(K_{c}+K_{r}\overset{\text{2}}{\underset{p}{r}}\right)}{\overset{\text{2}}{\underset{eq}{J}}G^{\text{2}}}$$ (48)

$$b_{\text{4}}=-\frac{K_{c}+K_{r}\overset{\text{2}}{\underset{p}{r}}}{J_{eq}G^{\text{2}}}+\frac{\overset{\text{2}}{\underset{eq0}{B}}}{\overset{\text{2}}{\underset{eq}{J}}}-\frac{\overset{\text{2}}{\underset{t}{K}}}{J_{eq}L_{m}}$$ (49)

$$b_{\text{5}}=-\frac{B_{eq0}K_{t}}{\overset{\text{2}}{\underset{eq}{J}}}-\frac{K_{t}R_{m}}{J_{eq}L_{m}}$$ (50)

$$b_{\text{6}}=\frac{B_{eq0}}{\overset{\text{2}}{\underset{eq}{J}}}$$ (51)

The total disturbances (*d*) consist of the effects of state variables, observed gains, parameter uncertainties, and nonlinearities, as defined in (52).


$$\hat{d}=\sum_{i=1}^{\text{6}}b_{i}{\hat{x}}_{i}+\sum_{i=1}^{\text{6}}a_{i}\lambda_{i}+{\dot{\lambda }}_{\text{4}}+{\ddot{\lambda }}_{\text{3}}$$ (52)

Taking the third derivative of the control error and combining it with (42), we get (53).


$$\overset{...}{e}={\overset{...}{v}}_{\text{1}}-{\overset{...}{\hat{x}}}_{\text{3}}={\overset{...}{v}}_{\text{1}}-\overset{...}{y}$$ (53)

A control law based on the disturbance rejection mechanism is proposed in (54), where *u*_0_ is base control input.


$$u=\frac{u_{\text{0}}-\hat{d}}{b}$$ (54)

A sliding surface (*s*) is proposed in (55), where *k*_1_ and *k*_2_ are positive coefficients selected to ensure Hurwitz stability. By taking the derivative of the sliding surface and combining it with (53), (56) is obtained.


$$s=\ddot{e}+k_{\text{1}}\dot{e}+k_{\text{2}}e$$ (55)

$$\dot{s}={\overset{...}{v}}_{\text{1}}-\overset{...}{y}+k_{\text{1}}\ddot{e}+k_{\text{2}}\dot{e}$$ (56)

The base control input is proposed in (57) with *k*_3_ being a positive coefficient.


$$u_{\text{0}}={\overset{...}{v}}_{\text{1}}+k_{\text{1}}\ddot{e}+k_{\text{2}}\dot{e}+k_{\text{3}}\text{tanh}\left(s\right)$$ (57)

Combining (45), (52), (54), and (57), the control input is rewritten as (58).


$$u=\frac{J_{eq}L_{m}}{K_{t}}\left[{\overset{...}{v}}_{\text{1}}+k_{\text{1}}\ddot{e}+k_{\text{2}}\dot{e}+k_{\text{3}}\text{tanh}\left(s\right)-\sum_{i=1}^{\text{6}}b_{i}{\hat{x}}_{i}-\sum_{i=1}^{\text{6}}a_{i}\lambda_{i}-{\dot{\lambda }}_{\text{4}}-{\ddot{\lambda }}_{\text{3}}\right]$$ (58)

**Fig 3:**
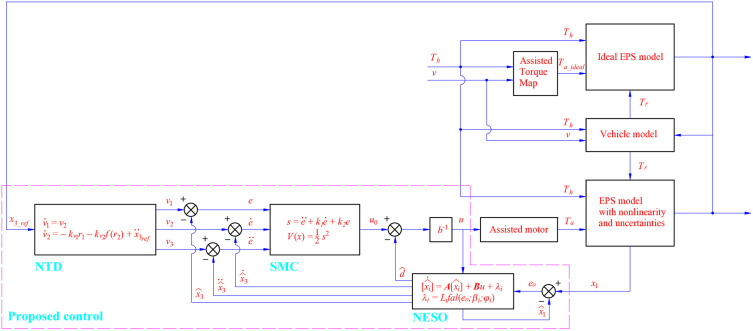
Proposed control scheme.

The structure of the proposed control is illustrated in Fig 3.

### Stability proofs

In this work, the observed parameters (*L*_*i*_) are determined using the pole placement method to guarantee the stability of the proposed NESO. The first Lyapunov candidate function, which represents the NTD, is introduced in (59). By taking the derivative of *V*_1_(*x*) and combining it with (31) and (32), (60) is obtained. The coefficients *k*_*v*1_ and *k*_*v*2_ are chosen to be positive, ensuring that *V*_1_(*x*) is positive definite and its derivative is negative definite for all nonzero *x*. In conclusion, the proposed NTD is stable according to the Lyapunov theory.


$$V_{\text{1}}\left(x\right)=\frac{\text{1}}{\text{2}}k_{v1}\overset{\text{2}}{\underset{\text{1}}{r}}+\frac{\text{1}}{\text{2}}\overset{\text{2}}{\underset{\text{2}}{r}}>0\forall x\neq 0$$ (59)

$${\dot{V}}_{\text{1}}\left(x\right)=k_{v1}r_{\text{1}}r_{\text{2}}+r_{\text{2}}\left[-k_{v1}r_{\text{1}}-k_{v2}\frac{r_{\text{2}}}{\sqrt{\overset{\text{2}}{\underset{\text{2}}{r}}+\theta^{\text{2}}}}\right]=-k_{v2}\frac{\overset{\text{2}}{\underset{\text{2}}{r}}}{\sqrt{\overset{\text{2}}{\underset{\text{2}}{r}}+\theta^{\text{2}}}}<0\forall x\neq 0$$ (60)

The second Lyapunov candidate function, which represents the proposed control, is presented in (61). By taking the derivative of *V*_2_(*x*) and combining it with (45), (54), (56), and (57), (62) is obtained. The coefficient *k*_3_ is selected to be positive. Consequently, *V*_2_(*x*) is positive definite and its derivative is negative definite for all nonzero *x*. In conclusion, the proposed control is stable in the sense of Lyapunov.


$$V_{\text{2}}\left(x\right)=\frac{\text{1}}{\text{2}}s^{\text{2}}>0\forall x\neq 0$$ (61)

$$\begin{array}{c} {\dot{V}}_{\text{2}}\left(x\right)=s\dot{s}=s\left({\overset{...}{v}}_{\text{1}}-\overset{...}{y}+k_{\text{1}}\ddot{e}+k_{\text{2}}\dot{e}\right)\\ =s\left[{\overset{...}{v}}_{\text{1}}-\left(\hat{d}+bu\right)+k_{\text{1}}\ddot{e}+k_{\text{2}}\dot{e}\right]\\ =s\left[{\overset{...}{v}}_{\text{1}}-u_{\text{0}}+k_{\text{1}}\ddot{e}+k_{\text{2}}\dot{e}\right]\\ =s\left[{\overset{...}{v}}_{\text{1}}-\left({\overset{...}{v}}_{\text{1}}+k_{\text{1}}\ddot{e}+k_{\text{2}}\dot{e}+k_{\text{3}}\text{tanh}\left(s\right)\right)+k_{\text{1}}\ddot{e}+k_{\text{2}}\dot{e}\right]\\ =-k_{\text{3}}s\text{tanh}\left(s\right)<0\forall x\neq 0 \end{array}$$ (62)

## 3. Simulation and result

The numerical simulations are conducted to evaluate the effectiveness of the proposed control strategy. The results are benchmarked against several controllers, namely ADRC, PID, and SMC.

**SMC:** The state variables are assumed to be directly measured by sensors without considering sensor noise. Moreover, the control law does not account for parameter uncertainties and system nonlinearities.

ADRC: The state variables are estimated using a Linear Extended State Observer (LESO), which inherently incorporates the effects of parameter uncertainties and nonlinear dynamics.

**PID:** The control law is formulated based on a simple proportional–integral–derivative mechanism.

The steering input is a periodic sine function, as illustrated in (63). The vehicle speed is maintained at *v* = 20 km/h under two testing scenarios. In the first case, the effects of nonlinear friction and parameter uncertainties are neglected. In the second case, both effects are fully considered, with the friction coefficient denoted as *τ*_*m*_ and parameter uncertainties set at 10%.


$$T_{h}=9\;sin\;2t$$ (63)

The vehicle specifications are listed in Table 1.

**Table 1:** Vehicle specifications.

Symbol	Unit	Value	Symbol	Unit	Value	Symbol	Unit	Value
*J*_*c*_	kgm^2^	0.055	*B*_*c*_	Nms/rad	0.07	*l*_*n*_	m	0.3
*K*_*c*_	Nm/rad	140	*G*	–	19	*γ*_*k*_	°	10
*K*_*t*_	Nm/A	0.06	*L*_*m*_	H	0.005	*l*_*f*_	m	1.150
*R*_*m*_	Ω	0.6	*B*_*m*_	Nms/rad	0.0050	*l*_*r*_	m	1.650
*B*_*r*_	Ns/m	4350	*M*_*r*_	kg	28.5	*γ*_*c*_	°	3
*r*_*p*_	m	0.015	*J*_*m*_	kgm^2^	0.0004	*m*	kg	1480
*τ*_*m*_	Nm	0.1	*l*_*c*_	m	0.03	*J*_*ψ*_	kgm^2^	3030

### 3.1. The first case without nonlinearity and parametric uncertainties

**Fig 4:**
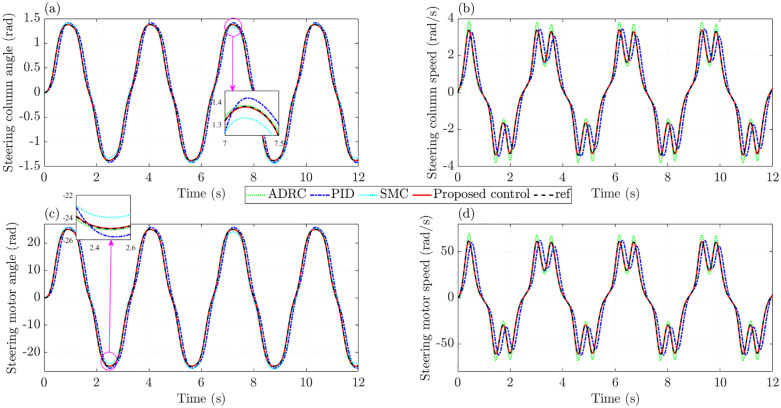
Actuator dynamics without nonlinearity and parametric uncertainties. **(a)** Steering column angle; **(b)** Steering column speed; **(c)** Steering motor angle; **(d)** Steering motor speed.

In the first case, the effects of nonlinearity and parametric uncertainties are neglected; therefore, the practical model closely resembles the ideal one. The variations in actuator dynamics are illustrated in Fig 4. Fig 4a presents the change in the steering column angle over time. A closer examination of this subplot shows that the PID controller yields an RMS tracking error of 14.240%, approximately 3.3 times higher than that of the SMC controller (4.299%). By contrast, ADRC achieves a reduced RMS tracking error of 1.660%, while the proposed control further improves accuracy with a remarkably low error of only 0.165%. Regarding the steering column speed shown in Fig 4b, the most significant tracking error is seen with the PID controller, reaching 0.687 rad/s (33.444%). Even without accounting for nonlinear friction and parameter uncertainties, the RMS tracking error produced by ADRC remains as high as 8.472%, considerably larger than that of SMC (1.682%). The simulation results demonstrate that the proposed controller performs better with tracking error not exceeding 0.004 rad/s (0.179%).

When the steering motor angle is taken as the controlled state, the tracking error under the proposed controller remains extremely small, with an RMS value of only 0.172%. Once the proposed method is replaced, the error rises noticeably to 1.666% with ADRC, 4.504% with SMC, and as high as 14.787% with PID (Fig 4c). A closer look at Fig 4d shows that the PID controller fails to follow the reference trajectory, while ADRC produces a slight overshoot. In contrast, such drawbacks are almost completely removed when the proposed controller controls the system.

**Fig 5:**
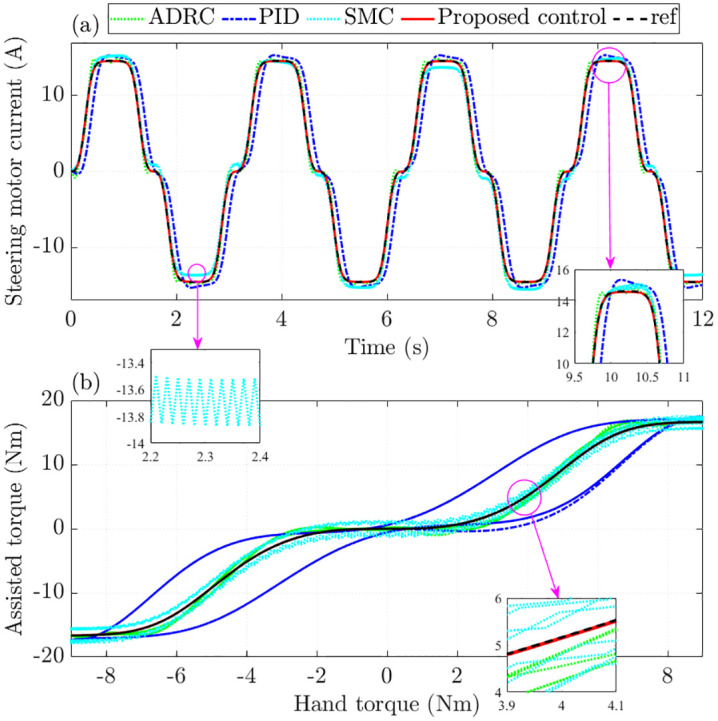
Assisted performance without nonlinearity and parametric uncertainties. **(a)** Steering motor current; **(b)** Assisted torque.

The performance of the assisted motor is evaluated through the steering motor current and the assisted torque, as illustrated in Fig 5. Simulation results in Fig 5a indicate that the RMS tracking error of the motor current can reach as high as 25.117% under PID control. Although this error is reduced to 6.884% with SMC, the presence of chattering degrades control performance and accelerates mechanical wear. By contrast, the proposed control substantially improves, lowering the RMS tracking error to only 0.263%, significantly lower than that obtained with ADRC (4.857%). Moreover, the phase lag seen with PID and the chattering effect associated with SMC are eliminated under the proposed scheme. Regarding Total Harmonic Distortion (THD), the motor current records 12.210% with PID and 13.710% with the proposed control. Nevertheless, ADRC results in a considerably higher THD of 16.610%, leading to notable energy losses.

A closer inspection of Fig 5b indicates that the assist-torque produced by the proposed control closely follows the reference, whereas the alternative controllers exhibit noticeably larger errors.

**Fig 6:**
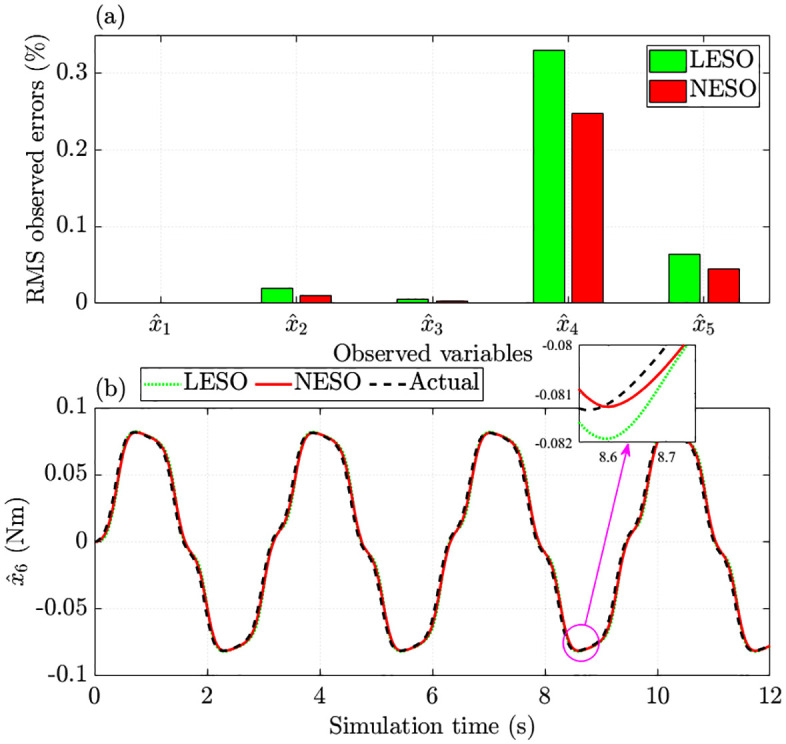
Observed errors without nonlinearity and parametric uncertainties. **(a)** RMS observed errors; **(b)** Augmented variable error.

Fig 6a compares the RMS errors observed by the LESO (ADRC) with those of the NESO (proposed control). The observed error for the steering column angle is effectively zero after rounding. Regarding the remaining states, LESO yields higher errors than NESO: the RMS error in steering motor speed is 0.330% with LESO versus 0.248% with NESO, and for motor current, the corresponding errors are 0.064% and 0.044%, respectively.

As shown in Fig 6b, the observed error of the augmented variable under the proposed controller is only 6.538%, lower than the 8.248% recorded with ADRC. This result demonstrates that the proposed control scheme equipped with NESO performs better in estimating state variations and disturbances.

**Fig 7:**
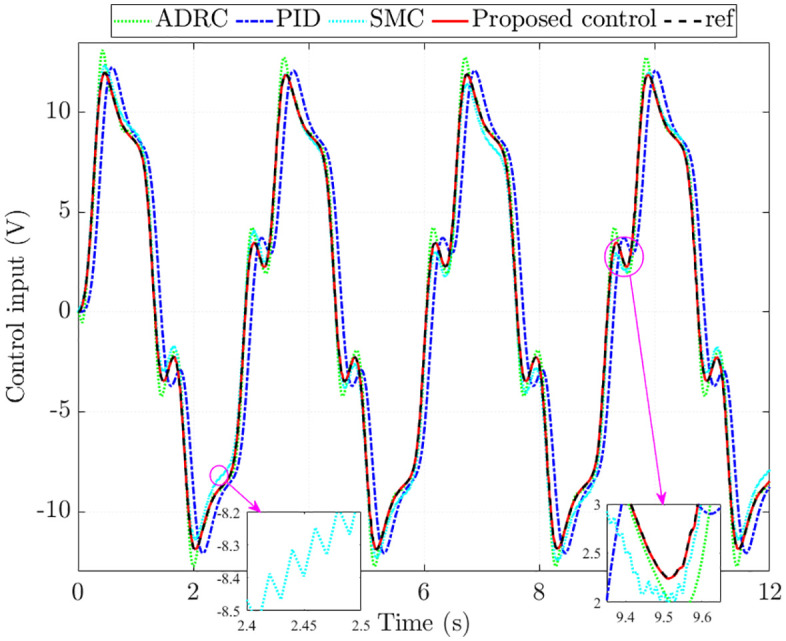
Control input without nonlinearity and parametric uncertainties.

Fig 7 illustrates the control input. The simulation results indicate that the voltage signal generated by the proposed controller closely tracks the reference, with an RMS error of merely 0.249%. In contrast, the corresponding errors are significantly larger for the other methods: 6.330% for ADRC, 28.845% for PID, and 5.894% for SMC. Chattering is clearly seen in the SMC input, which compromises system performance and accelerates mechanical wear. ADRC exhibits a slight overshoot, while PID is affected by a noticeable phase delay. These drawbacks are completely eliminated when the proposed scheme replaces the conventional controllers.

The computed results in this case indicate that the total energy consumption under the proposed controller is only 985,420 J, slightly lower than that of SMC (992,332 J). In contrast, the energy usage of PID and ADRC reaches 1,012.678 J and 1,030.420 J, respectively. The simulation outcomes are summarized in Tables 2 and 3. It should be noted that all reported values have been rounded.

**Table 2:** Tracking and observed errors in the first case.

	Proposed control		ADRC		PID	SMC
	Tracking error (%)	Observed error (%)	Tracking error (%)	Observed error (%)	Tracking error (%)	Tracking error (%)
Steering column angle	0.165	0.000	1.660	0.000	14.240	4.299
Steering column speed	0.179	0.011	8.472	0.019	33.444	1.682
Steering motor angle	0.172	0.003	1.666	0.005	14.787	4.504
Steering motor speed	0.184	0.248	8.169	0.330	33.729	1.773
Motor current	0.263	0.044	4.857	0.064	25.117	6.884
Augmented variable		6.538		8.248		

**Table 3:** Actuator performance in the first case.

	Proposed control	ADRC	PID	SMC
Total harmonic distortion (%)	13.710	16.610	12.210	13.730
Control input error (%)	0.249	6.330	28.845	5.894
Total energy consumption (J)	985.420	1030.420	1012.678	992.332

### 3.2. The second case with nonlinearity and parametric uncertainties

**Fig 8:**
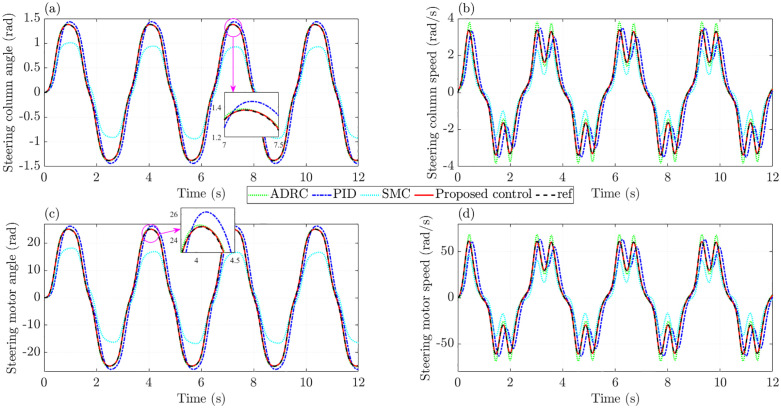
Actuator dynamics with nonlinearity and parametric uncertainties. **(a)** Steering column angle; **(b)** Steering column speed; **(c)** Steering motor angle; **(d)** Steering motor speed.

In the second case, the effects of nonlinear friction and parameter uncertainties are introduced, while other conditions, such as steering input and vehicle speed, remain unchanged. The aim is to assess the performance of the proposed controller under these adverse factors. Fig 8 illustrates the variations in actuator dynamics influenced by nonlinear friction and parameter uncertainties. As shown in Fig 8a, the RMS tracking error of PID increases to 15.777%, whereas that of SMC rises sharply to 32.347%. In contrast, only minor changes are seen in ADRC and the proposed control, with RMS tracking errors of 1.811% and 0.704%, respectively. Regarding the steering column speed, the RMS tracking error increases only slightly when regulated by the proposed control. At the same time, a considerable deterioration is evident when the system is controlled solely by conventional SMC (Fig 8b).

Fig 8c shows that, even in the presence of nonlinear friction and parameter uncertainties, the steering motor angle under the proposed controller remains extremely close to the reference signal, with the RMS error limited to 0.736%. By comparison, conventional controllers produce much larger errors and a noticeable loss of performance. The trend seen in steering motor speed is similar to that of the steering column speed, but with higher magnitudes (Fig 8d).

**Fig 9:**
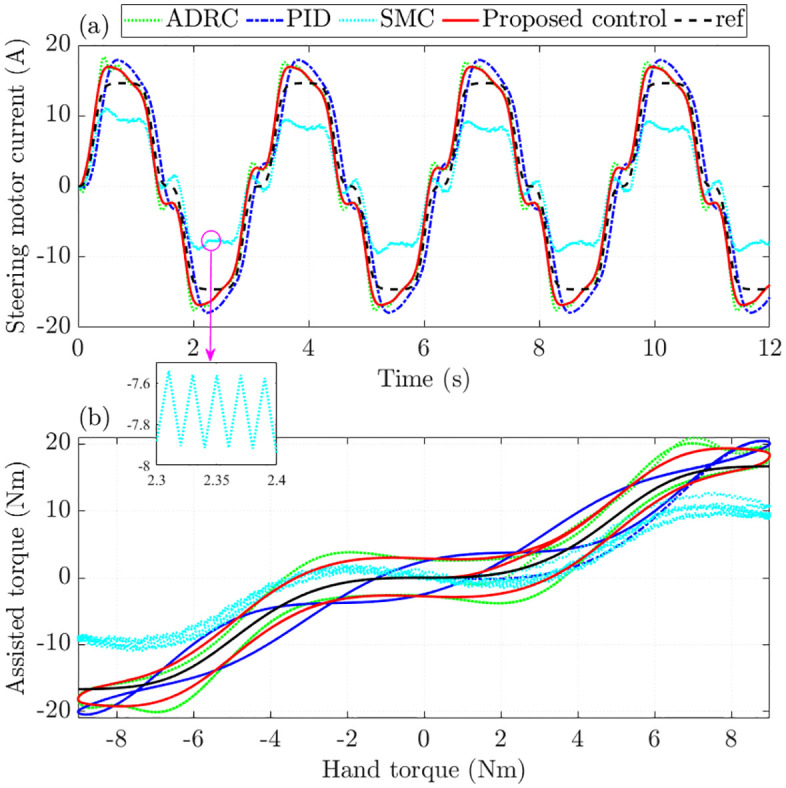
Assisted performance with nonlinearity and parametric uncertainties. **(a)** Steering motor current; **(b)** Assisted torque.

When parameter uncertainties and nonlinear disturbances are considered, standard SMC’s effectiveness drops considerably, as illustrated in Fig 9. In contrast, the proposed control maintains more accurate tracking than ADRC and PID, a result confirmed by the RMS error values. Furthermore, the strong chattering effect associated with SMC disappears entirely once the proposed method is applied.

**Fig 10:**
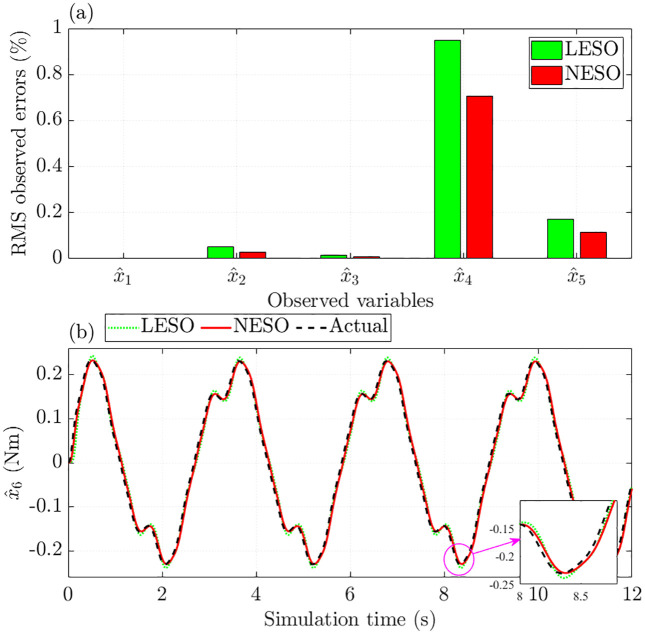
Observed errors with nonlinearity and parametric uncertainties. **(a)** RMS observed errors. **(b)** Augmented variable error.

As illustrated in Fig 10a, the observed errors produced by NESO are consistently lower than those of LESO. In particular, the augmented variable’s RMS observed error is 7.125% with NESO, compared to 8.784% with LESO (Fig 10b). These findings indicate that the proposed control scheme provides more accurate state estimation than linear observers, even under significant uncertainties and nonlinearities.

In this case, the THD value of the proposed control remains unchanged at 13.710%, whereas the corresponding values for ADRC and SMC increase considerably to 17.160% and 24.110%, respectively.

The control input generated by SMC fails to track the reference signal, primarily because its control law does not account for the influence of nonlinear friction and parameter uncertainties. In addition, chattering is clearly seen in the voltage signal, which leads to a considerable reduction in system performance. By contrast, the proposed control demonstrates superior capability in regulating the system, keeping the tracking error to around 15% even under the combined effects of nonlinear friction and parameter variations.

**Fig 11:**
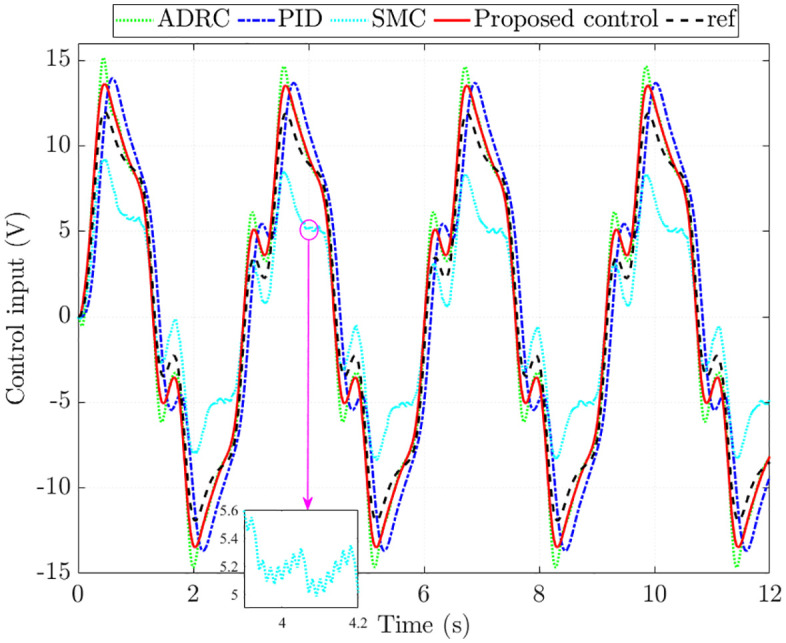
Control input with nonlinearity and parametric uncertainties.

The simulation results further show that the total energy consumption of the proposed control is 1194.581 J (Fig 11), lower than that of ADRC (1248.965 J) and PID (1263.312 J). SMC consumes less energy in this case since its control input does not correctly follow the reference signal.

The simulation results for the second case (rounded) are listed in Tables 4 and 5.

**Table 4:** Tracking and observed errors in the second case.

	Proposed control		ADRC		PID	SMC
	Tracking error (%)	Observed error (%)	Tracking error (%)	Observed error (%)	Tracking error (%)	Tracking error (%)
Steering column angle	0.704	0.000	1.811	0.000	15.777	32.347
Steering column speed	0.673	0.030	9.034	0.052	34.410	31.472
Steering motor angle	0.736	0.009	1.821	0.014	16.403	33.842
Steering motor speed	0.695	0.705	8.722	0.949	34.776	32.844
Motor current	18.510	0.113	20.927	0.171	21.366	41.459
Augmented variable		7.125		8.784		

**Table 5:** Actuator performance in the second case.

	Proposed control	ADRC	PID	SMC
Total harmonic distortion (%)	13.710	17.160	11.190	24.110
Control input error (%)	15.766	19.032	27.992	37.230
Total energy consumption (J)	1194.581	1248.965	1263.312	381.674

## 4. Conclusion

This article presents the design of the robust control approach for the EPS system that explicitly accounts for the effects of nonlinear friction and parametric uncertainties. The proposed controller, developed by integrating smooth SMC and NADRC, aims to reduce tracking errors and minimize energy consumption. Simulation results confirm that the outputs closely follow the reference signals even under nonlinear and uncertain conditions. Furthermore, overshooting, phase delay, and chattering are effectively eliminated when the proposed strategy controls the system.

Despite its effectiveness in EPS control, several drawbacks remain. Firstly, the observed error of the augmented variable is relatively large. Secondly, the tracking error of the motor current is significant under adverse conditions. Finally, the control parameters should be tuned adaptively to improve system performance across different operating conditions. These issues are expected to be addressed in future work.

## Supporting information

S1 File All relevant data is contained within the manuscript and Supporting Information File S1. (DOCX)
