# Supplementary material for: An integrated SMC–NADRC robust control approach for electric power steering systems considering nonlinear friction and parametric uncertainties
Source: PLoS One. 2026 Apr 9;21(4):e0346332. doi: 10.1371/journal.pone.0346332 (PMC13065078; doi:10.1371/journal.pone.0346332)
Supplement: S1 File — (DOCX) [file pone.0346332.s001.docx]

Table 1. Vehicle specifications.

| **Symbol** | **Unit** | **Value** | **Symbol** | **Unit** | **Value** | **Symbol** | **Unit** | **Value** |
| --- | --- | --- | --- | --- | --- | --- | --- | --- |
| *J_c_* | kgm^2^ | 0.055 | *B_c_* | Nms/rad | 0.07 | *l_n_* | m | 0.3 |
| *K_c_* | Nm/rad | 140 | *G* | - | 19 | *γ_k_* | ° | 10 |
| *K_t_* | Nm/A | 0.06 | *L_m_* | H | 0.005 | *l_f_* | m | 1.150 |
| *R_m_* | Ω | 0.6 | *B_m_* | Nms/rad | 0.0050 | *l_r_* | m | 1.650 |
| *B_r_* | Ns/m | 4350 | *M_r_* | kg | 28.5 | *γ_c_* | ° | 3 |
| *r_p_* | m | 0.015 | *J_m_* | kgm^2^ | 0.0004 | *m* | kg | 1480 |
| *τ_m_* | Nm | 0.1 | *l_c_* | m | 0.03 | *J_ψ_* | kgm^2^ | 3030 |

Table 2. Tracking and observed errors in the first case.

|  | **Proposed control** | | **ADRC** | | **PID** | **SMC** |
| --- | --- | --- | --- | --- | --- | --- |
|  | Tracking error (%) | Observed error (%) | Tracking error (%) | Observed error (%) | Tracking error (%) | Tracking error (%) |
| Steering column angle | 0.165 | 0.000 | 1.660 | 0.000 | 14.240 | 4.299 |
| Steering column speed | 0.179 | 0.011 | 8.472 | 0.019 | 33.444 | 1.682 |
| Steering motor angle | 0.172 | 0.003 | 1.666 | 0.005 | 14.787 | 4.504 |
| Steering motor speed | 0.184 | 0.248 | 8.169 | 0.330 | 33.729 | 1.773 |
| Motor current | 0.263 | 0.044 | 4.857 | 0.064 | 25.117 | 6.884 |
| Augmented variable |  | 6.538 |  | 8.248 |  |  |

Table 3. Actuator performance in the first case.

|  | **Proposed**  **control** | **ADRC** | **PID** | **SMC** |
| --- | --- | --- | --- | --- |
| Total harmonic distortion (%) | 13.710 | 16.610 | 12.210 | 13.730 |
| Control input error (%) | 0.249 | 6.330 | 28.845 | 5.894 |
| Total energy consumption (J) | 985.420 | 1030.420 | 1012.678 | 992.332 |

Table 4. Tracking and observed errors in the second case.

|  | **Proposed control** | | **ADRC** | | **PID** | **SMC** |
| --- | --- | --- | --- | --- | --- | --- |
|  | Tracking error (%) | Observed error (%) | Tracking error (%) | Observed error (%) | Tracking error (%) | Tracking error (%) |
| Steering column angle | 0.704 | 0.000 | 1.811 | 0.000 | 15.777 | 32.347 |
| Steering column speed | 0.673 | 0.030 | 9.034 | 0.052 | 34.410 | 31.472 |
| Steering motor angle | 0.736 | 0.009 | 1.821 | 0.014 | 16.403 | 33.842 |
| Steering motor speed | 0.695 | 0.705 | 8.722 | 0.949 | 34.776 | 32.844 |
| Motor current | 18.510 | 0.113 | 20.927 | 0.171 | 21.366 | 41.459 |
| Augmented variable |  | 7.125 |  | 8.784 |  |  |

Table 5. Actuator performance in the second case.

|  | **Proposed**  **control** | **ADRC** | **PID** | **SMC** |
| --- | --- | --- | --- | --- |
| Total harmonic distortion (%) | 13.710 | 17.160 | 11.190 | 24.110 |
| Control input error (%) | 15.766 | 19.032 | 27.992 | 37.230 |
| Total energy consumption (J) | 1194.581 | 1248.965 | 1263.312 | 381.674 |
